# Development and application of a quantitative real-time PCR method for detection of Decapod iridescent virus 1

**DOI:** 10.3389/fmicb.2024.1472782

**Published:** 2024-09-19

**Authors:** Fu-Rong Zhao, Yang Liu, Qin Zheng, Yan-Ge Zhang, Yijuan Han, Dong-Hui Zhou, Gui-Chao Ma, Wei Wang, Jianming Chen

**Affiliations:** ^1^Fujian Key Laboratory on Conservation and Sustainable Utilization of Marine Biodiversity, Fuzhou Institute of Oceanography, Minjiang University, Fuzhou, China; ^2^Key Laboratory of Fujian-Taiwan Animal Pathogen Biology, College of Animal Sciences, Fujian Agriculture and Forestry University, Fuzhou, China

**Keywords:** DIV1, SYBR Green I^®^, real-time PCR, MCP gene, detection method

## Abstract

As a newly discovered virus, Decapoda iridovirus 1 (DIV1) can cause a mortality rate of up to 100% in crustaceans, leading to huge economic losses. At present, there is no effective prevention and control measures for this disease. In the present study, the specific primers targeting highly conserved regions of MCP gene were designed, and then a quantitative real-time PCR method was established. The results indicate that DIV1 quantitative real-time PCR established has good specificity and does not cross react with other pathogens including white spot syndrome virus (WSSV), infectious subcutaneous and hematopoietic necrosis virus (IHHNV) and *Vibrio parahaemolyticus* induced acute hepatopancreatic necrosis disease (VpAHPND). The real-time PCR was capable of detecting DIV1 DNA at a minimum concentration of 10 copies/μL within 34 cycles. The method has good repeatability, with intra group and inter group coefficients of variation both less than 2%. Thirty-two clinical samples were assessed using both the real-time PCR and conventional PCR. The results shown real-time PCR we established are more sensitive than conventional PCR. In conclusion, this method has strong specificity, stable repeatability, and high sensitivity, providing technical support for clinical diagnosis, epidemiology investigation and monitoring of DIV1.

## 1 Introduction

Decapod iridescent virus 1 (DIV1) is first identified in 2014 and an emerging cytoplasmic nucleocytoplasmic large DNA virus containing linear double-stranded DNA (Arulmoorthy et al., 2022). Its structure exhibits icosahedral symmetry, appearing hexagonal when viewed in planar form. The genome of DIV1 is 165,695 bp including a total of 178 open reading frames (ORF) (Williams et al., 2005; Chinchar et al., 2009; Li et al., 2017). Qiu et al. (2017) discovered a new type of iridovirus in the Vannamei shrimp from Zhejiang province and named it Shrimp hemocyte iridescent virus (SHIV) in 2014. In 2016, Xu et al. (2016) identified a new type of iridovirus in the diseased material of the red claw crayfish from Fujian province and named it *Cherax quadricarinatus* iridovirus (CQIV). It was determined that the genomic homology between SHIV and CQIV is 99.97% by bioinformation analysis. In 2019, the International Committee on Taxonomy of Viruses (ICTV) unified the names SHIV and CQIV to DIV1, categorizing it as a new genus within the Iridoviridae family, namely the Decapod iridescent virus genus (OIE, 2023).

DIV1 is characterized by a broad host range, long incubation period, rapid transmission rate, and high mortality rate causing decapod crustaceans. Horizontal transmission is the main route of transmission for DIV1, including cannibalism among the same species, consumption of bait carrying the virus, and mixed culture of healthy animals with sick animals (Lightner, 2011). DIV1 has been reported to infect a variety of crustaceans such as the *Cherax quadricarinatus*, *Litopenaeus vannamei*, *Macrobrachium nipponense*, *Macrobrachium rosenbergii*, *Procambarus clarkii*, *Penaeus monodon*, *Exopalaemon carinicauda* and *Portunus trituberculatus* (Xu et al., 2016; Qiu et al., 2017; He et al., 2021), mainly infecting their hematopoietic tissue, lymphatic tissue, gills, and hepatopancreas. Clinical symptoms of Shrimp infected with DIV1 include weakness, anorexia, empty intestines and stomach, pale and atrophied hepatopancreas, and reddening of the shrimp body, followed by mass mortality. It has been reported that the three-spined mud crab, Chinese mitten crab, and thick-legged mud crab can also be infected with DIV1 under artificial infection or breeding conditions, showing symptoms of white gill filaments and edema (Qin et al., 2023). Since 2014, infection with DIV1 has been reported in several provinces in China, including Zhejiang, Guangdong and Hebei provinces. Subsequently in 2017 and 2018, target surveillance revealed that DIV1 was detected in 11 of 16 provinces in China. In addition, DIV1 was first detected in shrimp and crayfish farms in Taiwan, China in 2020 (Yiping et al., 2023). There have been reports of DIV1 from Thailand at a very low prevalence, but this is yet to be officially confirmed (OIE, 2023). In 2020, DIV1 was discovered and isolated in wild *Penaeus monodon* (without any clinical signs of disease) in the Indian Ocean (Srisala et al., 2021). The widespread prevalence of DIV1 in aquaculture not only causes significant economic losses to the industry, but also poses a great threat to its healthy development.

Currently, there are no effective measures for prevention and control to DIV1, detection and monitoring are the key measures to control the spread of the virus. The diagnostic methods for DIV1 that have been established include clinical diagnosis, histopathological diagnosis, and molecular biological diagnosis. Crustaceans infected with DIV1 exhibit no clinical symptoms, so accurate identification and diagnosis cannot rely solely on clinical diagnosis. Histopathological diagnosis has been complex to operate, which is not conducive to large-scale clinical diagnosis and can only serve as an auxiliary examination method. At present, molecular biological diagnosis is the most important method for the detection of DIV1. The World Organization for Animal Health (OIE) recommends using nested PCR and real-time PCR to DIV1 detection (Qiu et al., 2017, 2018, 2020). But nested PCR has issues such as false positives, operational difficulties, and susceptibility to contamination. Real-time PCR is the most sensitive and specific method for detecting and quantifying shrimp viruses. Nowadays commonly used real-time PCR approaches include probe-based and SYBR Green I-based methods. TaqMan probe-based real-time PCR is relatively expensive and troublesome because it requires a specific probe for virus detection. Therefore, this study designed specific primers for the target gene MCP of DIV1 and established a method based on SYBR Green I real-time PCR to DIV1 detecting and quantifying in *Cherax quadricarinatus*, which has the advantages of low cost, simplicity, strong specificity and high sensitivity. The method suitable for large-scale qualitative and quantitative testing for DIV1 in clinical samples, providing effective technical support for laboratory diagnosis, clinical testing, and prevention and control of DIV1.

## 2 Materials and methods

### 2.1 Animals and viruses

The *Cherax quadricarinatus* (red-claw crayfish) were purchased from a crayfish hatchery in Zhangzhou city, Fujian Province. These shrimps have been tested and confirmed not to be infected with common pathogens in laboratory, then use it for subsequent experiments. DNA samples of shrimps infected with DIV1, white spot syndrome virus (WSSV), infectious hypodermal and hematopoietic necrosis virus (IHHNV), and the bacterium *Vibrio parahaemolyticus* causing acute hepatopancreatic necrosis disease (VpAHPND) are preserved in our laboratory.

### 2.2 The primers design and synthesis of MCP of DIV1

Primers were designed using the highly conserved MCP of DIV1 (GenBank accession number: NC_040612.1) as a template by the Primer Premier 5.0 software and verified for good specificity through Primer-BLAST of NCBI. These primers were manufactured by Sangon Biotech (Shanghai) Co., Ltd. (Table 1).

**Table 1:** The primer sequences for conventional PCR and real-time PCR in the study.

Primers	Sequences from 5′ to 3′	Base number	Note
PCR-MCP-F	CCGTCCTCAACCCAAATC	435 bp	PCR
PCR-MCP-R	TGGCTTCACCTTCACCCT		
qPCR-MCP-F	TGATGACTGCCGATTACTTCTC	161 bp	Real-time PCR
qPCR-MCP-R	TTGGATACTCACATTGTTCAGGAT		

### 2.3 PCR amplification of MCP of DIV1

Total DNA was extracted from the muscle tissue of red-claw crayfish infected with DIV1 using a DNeasy Blood & Tissue kit (Qiagen, Germany) according to the manufacturer’s instructions. The DNA was used as a template to PCR amplification of the 435 bp MCP gene fragment using designed primers PCR-MCP-F/R. Briefly, after an initial denaturation step at 94°C for 5 min, denaturation at 94°C for 30 s, annealing at 57°C for 30 s, extension at 72°C for 1 min, for a total of 35 cycles; a final extension at 72°C for 5 min. After the PCR reaction, the amplified products were analyzed by electrophoresis on a 2% agarose gel, and their visualization was achieved under UV light. The PCR products showing the target fragment size were stored at 4°C.

### 2.4 Construction of recombinant standard plasmid

The purification of the PCR products were performed using the Gel Extraction Kit, then were sequenced by Sangon Biotech (Shanghai) Co., Ltd. Sequence was aligned and matched against the BLAST nucleotide database.^1^ The concentration of the purified PCR products was determined using a microplate spectrophotometer. It was ligated with the pMD19-T vector and then transformed into DH5α competent cells. Bacterial colonies that tested positive by PCR identification were sequenced, and those with correct sequencing results were scaled up for culture. Recombinant plasmids pMD19-T-MCP were extracted using the Plasmid Mini Kit (OMEGA). The concentration of the plasmid standards was measured using the Nano Drop 2000 spectrophotometer (Thermo Fisher, United States), and its copy number was calculated following by the formula: copy number (copies/μL) = 6.02 × 10^23^ × [(the concentration of plasmid standard (ng/μL) × 10^−9^)/(number of base pairs in the plasmid standard × 660)].

### 2.5 Establishment of standard curve for the quantitative real-time PCR

The plasmid standards were diluted in a ten-fold serial dilution (9.74 × 10^9^ to 9.74 × 10^2^ copies/μL), and the diluted plasmid standard was used as a template for quantitative real-time PCR (qPCR) amplification. Each concentration was set with three replicates, and negative control was included each independent test. PCR reaction program following as: pre-denaturation at 95°C for 30 s, denaturation at 95°C for 5 s, annealing and fluorescence signal collection at 60°C for 30 s, for a total of 40 cycles; 65°C for 5 s, fluorescence signal collection at 95°C for 5 s. The final standard curve is generated based on the CT value and the logarithm of standard copy number. After the completion of the detection, the results were analyzed by the standard curve and the melting peak curve.

### 2.6 Analysis of specificity, sensitivity, and reproducibility of qPCR method

#### 2.6.1 Specificity analysis

The established qPCR method was used to test the tissue DNA of red-claw crayfish infected with WSSV, IHHNV and VpAHPND, respectively. Healthy red-claw crayfish tissue DNA served as a negative control, and tissue DNA from red-claw crayfish infected with DIV1 served as a positive control to evaluate the specificity of the method.

#### 2.6.2 Sensitivity analysis

Ten-fold serial dilutions of the quantitative plasmids were prepared to be used as reference materials according to the copy number calculated, ranging from 3.339 × 10^1^ to 3.339 × 10^−8^ ng/μL. Using these 10 concentrations of plasmids samples as templates, conventional PCR and qPCR were performed using specific primers PCR-MCP-F/R and qPCR-MCP-F/R, respectively. The lowest detection copy numbers of the two methods were compared, and a negative control was set. The lowest detectable template concentration of the 2 methods was calculated and the difference in sensitivity was compared.

#### 2.6.3 Reproducibility analysis

The repeatability of the real-time PCR was assessed by intra- and inter-assays using the 10-fold serial dilutions of plasmid (9.74 × 10^3^, 9.74 × 10^4^, 9.74 × 10^5^ copies/μL). For the intra-assay test, three replicate samples from each dilution were tested in the same run. For the inter-assay test, each dilution of standard plasmids was tested in three independent runs to measure the test reliability or reproducibility regarding the mean CT-values with standard deviation (SD) and coefficient variation (CV). The coefficient of variation (CV) was evaluate the repeatability of the real-time PCR method. The CV is equal to the ratio of the standard deviation (SD) of the *C*_t_ value to the average of the *C*_t_ value.

### 2.7 Clinical samples application of the qPCR

To evaluate the clinical applicability of the qPCR method established in this study, 32 red clawed crayfish suspected with DIV1 infection samples collected from Zhangzhou city in Fujian province were test for DIV1. The DNA of these samples were extracted by using a Blood/Cell/Tissue Genomic DNA Extraction Kit (TIANGEN Biotech, Beijing, China) and subsequently used as a template for real-time PCR detection. Conventional PCR was performed on the genomic DNA of these samples using the primers PCR-MCP-F/R. The DIV1 positivity rate obtained from the two detection methods were compared.

## 3 Results

### 3.1 Construction of the plasmid standards for DIV1

**Figure 1:**
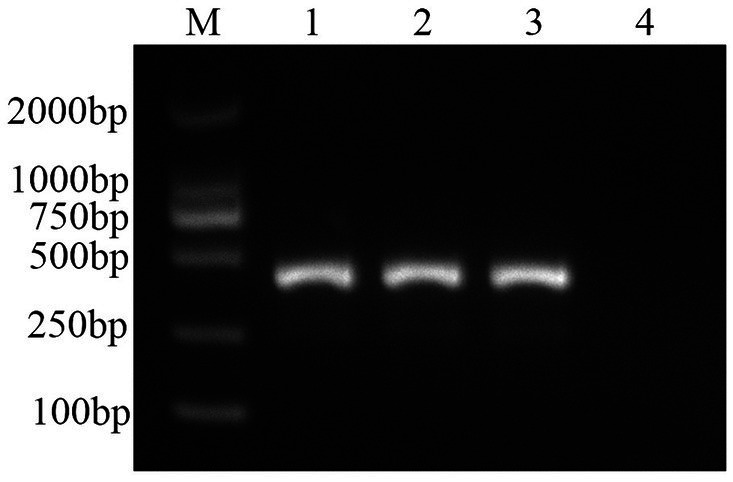
PCR amplified results of MCP gene fragments. The black arrow represents the size of the target gene. M: DL2000 DNA Marker; 1, 2: samples; 3: positive control; 4: negative control.

Using the DNA extracted from the muscle tissue of DIV1-infected red-claw crayfish as a template, PCR amplification was performed with the designed specific primers. A specific band of approximately 435 bp was successfully amplified, which was consistent with the size of the MCP gene fragment (Figure 1). The purified PCR product was sequenced. The sequence obtained identified to 100% homology with a segment of the MCP gene of DIV1 in GenBank. The amplified MCP gene fragment was inserted into the pMD19-T and then the sequencing results of the recombinant plasmid confirmed successful construction of the plasmid standard. The A_260_/_280_ value of the plasmid standard, measured using the microplate spectrophotometer was 1.99 with a concentration of 333.9 ng/μL. The calculated copy number was 9.74 × 10^10^ copies/μL.

### 3.2 Establishment of the standard curve

**Figure 2:**
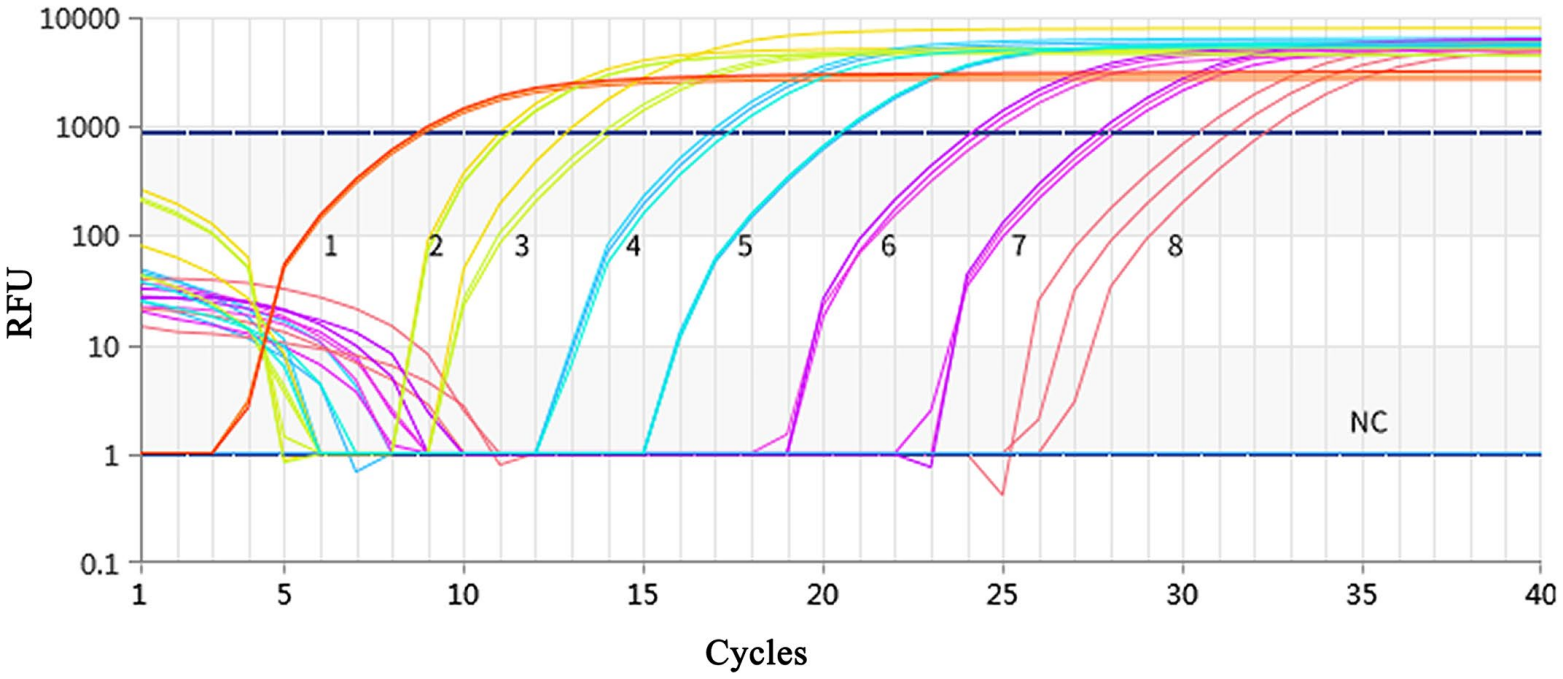
Fluorescence spectra for the standard curve. Fluorescence curves of different colors represent different concentrations of pMD19-T-MCP. The concentrations were 9.74 × 10^9^ to 9.74 × 10^2^ copies/μL from left to right, respectively. The NC represent negative control.

**Figure 3:**
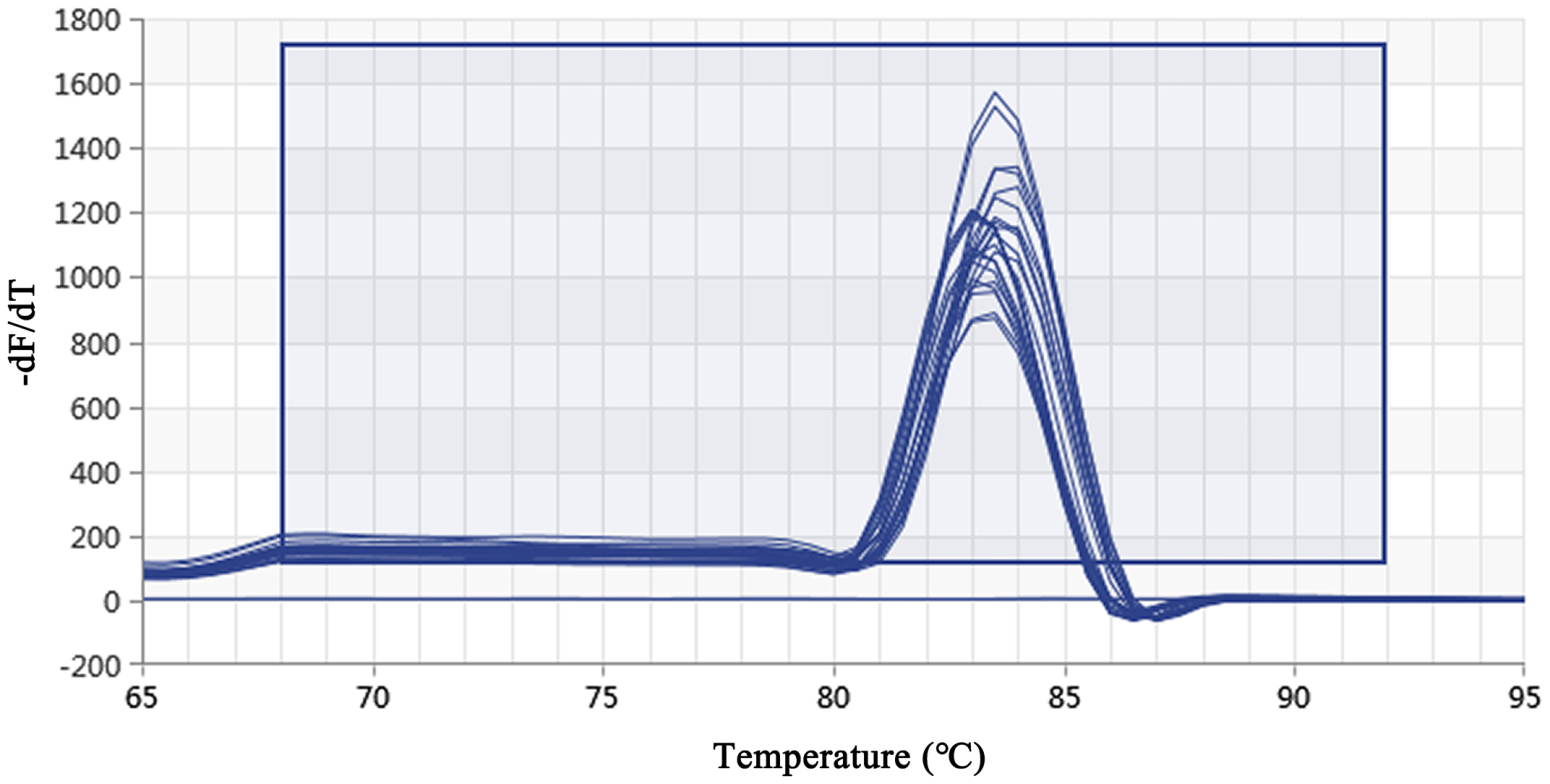
Real-time PCR melting peak curve based on DIV1. The melting peak curves of all samples showed a single peak, which indicated that the primers had excellent specificity.

**Figure 4:**
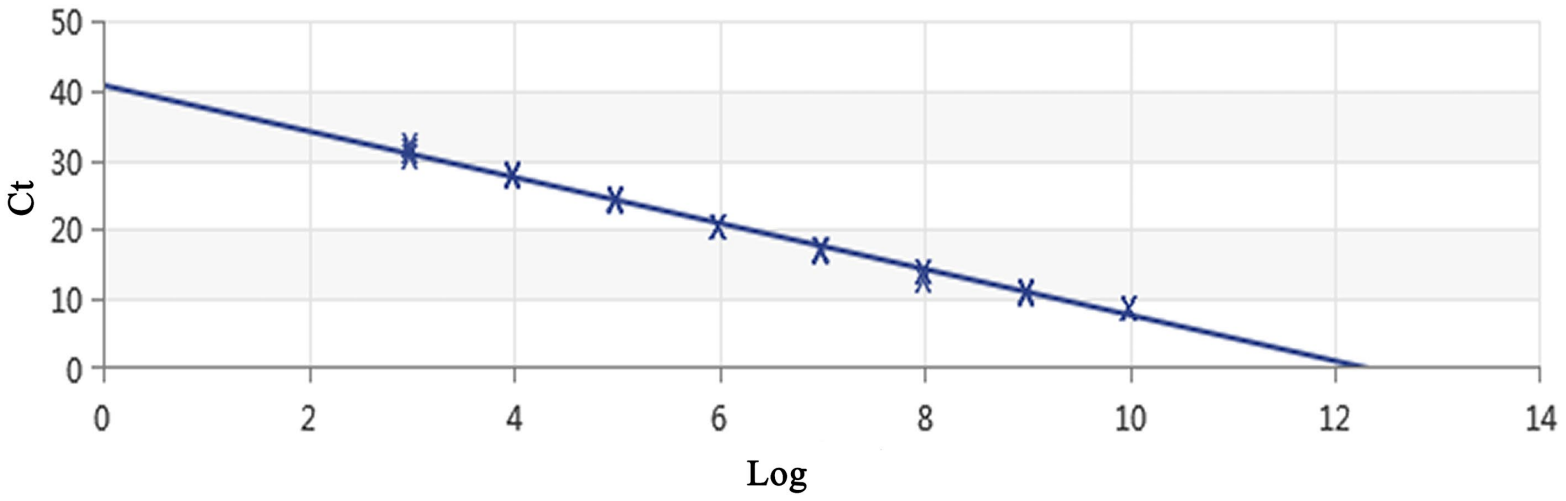
Establishment of the standard curve for MCP gene-based real-time PCR of DIV1. The 10-fold serial dilutions ranging from 9.74× 10^9^ to 9.74 × 10^2^ copies/μL of DNA plasmid were tested in the real-time PCR. Each point corresponds to the mean value of three replicates. The optimal standard formula was *Y* = −3.302*X* + 40.735, and the correlation coefficient was 0.995.

Using a plasmid standard with eight concentration gradients range from 9.74 × 10^9^ to 9.74 × 10^2^ copies/μL as a template for qPCR amplification, the results showed that the amplification curves of the MCP gene had good reproducibility. The logarithmic graph of relative fluorescence value for the standard curve extrapolation is shown in Figure 2. The melting curve analysis showed that all positive samples exhibited a single peak with a melting temperature of 83.40 ± 0.25°C (Figure 3). The equation of the standard curve is *Y* = −3.302*X* + 40.735 (where *X* is the logarithm of the copy number and *Y* is the *C*_t_ value), the correlation coefficient *R*^2^ is 0.995 and PCR amplification efficiency is 100.843% (Figure 4). The linearity between the logarithm of the copy number and the *C*_t_ value is excellent, and the single peak observed in the melting peak curve further confirms the high specificity of the primers and the successful establishment of this real-time PCR method. Meanwhile, no melting curves or dimer curves were observed in the negative control.

### 3.3 Analysis of specificity, sensitivity, and reproducibility of qPCR for DIV1

#### 3.3.1 Specificity analysis

**Figure 5:**
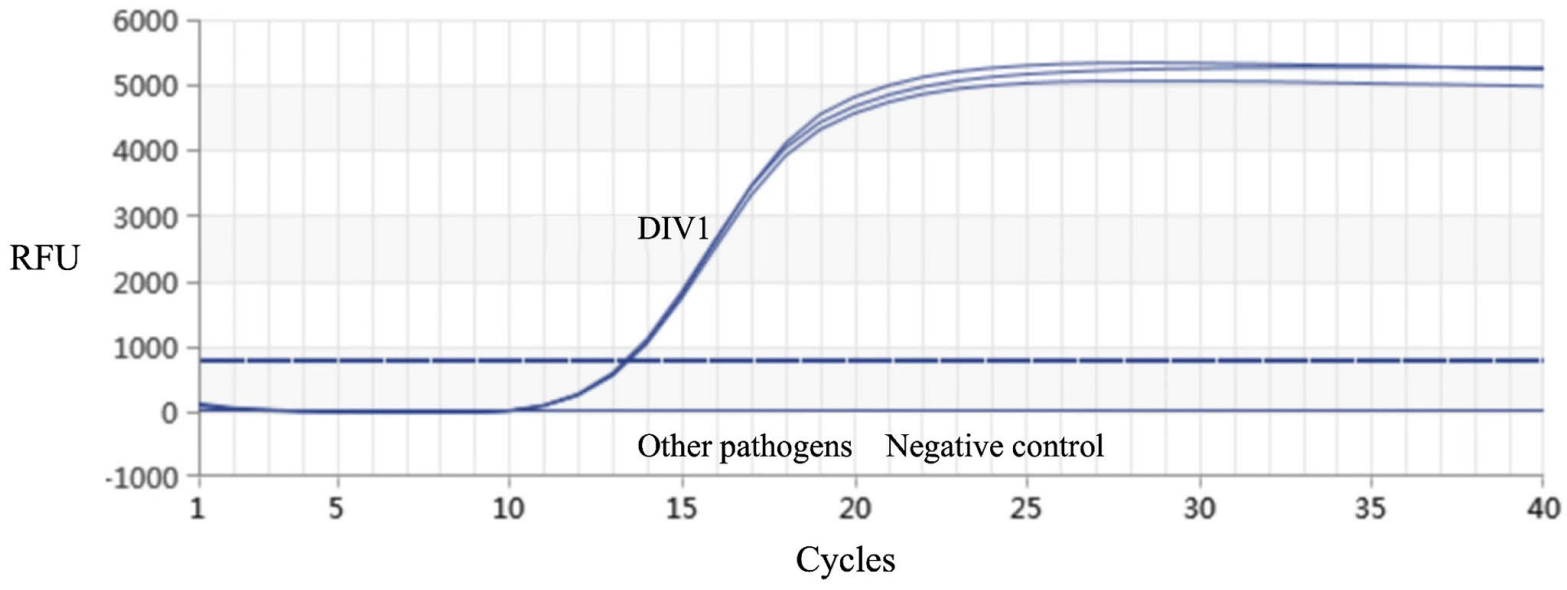
Real-time PCR amplification curves of DIV1 and other pathogens. Specificity analysis of the MCP gene-based real-time PCR. Only DIV1 showed a positive fluorescence signal, and no positive signal was observed with other pathogens.

Real-time PCR established in this study was used for detection on the muscle tissue DNA of healthy red-claw crayfish, as well as DNA samples of DIV1, WSSV, IHHNV, and VpAHPND, respectively. The results demonstrated that only the DIV1 sample produced a detectable amplification signal, while no specific amplification was observed for the other pathogens (Figure 5). These results indicated that the method exhibits high specificity.

#### 3.3.2 Sensitivity analysis

**Figure 6:**
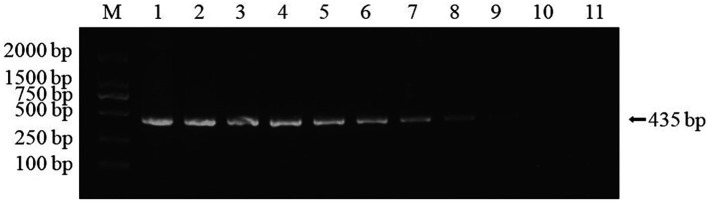
Sensitivity analysis of conventional PCR assay for detection of DIV1. The 10-fold serial dilutions ranging from 3.339 × 10^1^ to 3.339 × 10^−8^ ng/μL of quantitative plasmids were detected by conventional PCR. These diluted quantitative plasmids were used to perform the conventional PCR to obtain the expanded curve of the assays. The numbers 1 to 10 represented plasmid samples diluted to concentrations ranging from 3.339 × 10^1^ to 3.339 × 10^−8^ ng/μL, respectively. The numbers 11 represented the negative control.

**Figure 7:**
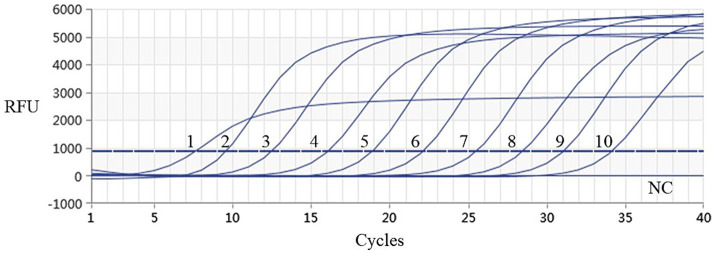
Sensitivity analysis results of real-time PCR assay for detection of DIV1. The numbers 1 to 10 represented the plasmid samples diluted to concentrations varying from 3.339 × 10^1^ to 3.339 × 10^−8^ ng/μL, respectively. These diluted quantitative plasmids samples were used to perform the real-time PCR to obtain the expanded curve of the assays. The NC represented the negative control.

Using the plasmids with 10 gradient diluted concentrations ranging from 3.339 × 10^1^ to 3.339 × 10^−8^ ng/μL (i.e., 9.74 × 10^9^–9.74 × 10^0^ copies/μL) as templates, both conventional PCR and qPCR were performed for DIV1. As shown in Figures 6, 7, conventional PCR was unable to detect the sample with a concentration below 9.74 × 10^2^ copies/μL, whereas the lowest copy number of the qPCR was 9.74 × 10^0^ copies/μL. These results demonstrate that the qPCR method established in this study is 100 times more sensitive than the conventional PCR method.

#### 3.3.3 Reproducibility analysis

Plasmid standards at three concentration (9.74 × 10^3^, 9.74 × 10^4^, 9.74 × 10^5^ copies/μL) were selected for the repeatability test. As shown in Table 2, the intra-group coefficients of variation ranging from 0.21 to 1.21%, while the inter-group coefficients of variation ranging from 0.98 to 1.71%. These result indicate that the qPCR method established in this study exhibits strong reproducibility.

**Table 2:** The results of the repeatability for real-time PCR based on DIV1 MCP gene.

Copy number/(copies μL^−1^)	Intra-group experiments		Inter-group experiments	
	*C*_t_	*CV*/%	*C*_t_	*CV*/%
9.74 × 10^5^	19.16 ± 0.04	0.21	19.17 ± 0.19	0.98
9.74 × 10^4^	23.40 ± 0.22	0.95	23.37 ± 0.40	1.71
9.74 × 10^3^	27.26 ± 0.33	1.21	27.22 ± 0.37	1.35

### 3.4 Application of the qPCR for DIV1

The qPCR developed in this study and conventional PCR was tested to the DIV1-suspected clinical samples, As shown in Table 3, the positive rate of DIV1 detection was 100% (32/32) by the qPCR, while the positive rate of DIV1 detection was 47% by the conventional PCR(17/32). These results shown the advantages of the qPCR detection method established in this study, which are more sensitive than conventional PCR.

**Table 3:** Detection results of clinical samples by real-time PCR and conventional PCR.

Detection methods	Results of the test			
	No. of positives	No. of negatives	Totals	Positive rate
Real-time PCR	32	0	32	100%
Conventional PCR	15	17	32	47%

## 4 Discussion

DIV1, as a newly discovered iridovirus in recent years, has not only been found in many coastal provinces of China, but also reported in shrimp in Thailand and the Indian Ocean etc. The hosts of DIV1 infected with have a variety of crustaceans including *Cherax quadricarinatus* (red-claw crayfish). The disease causing by DIV1 not only cause significant economic losses to aquaculture industry, but also pose a great threat to the global aquaculture industry. Since 2017, DIV1 has included in the list of monitored pathogens in the “National Aquatic Animal Disease Surveillance Program” in China. In 2020, DIV1 included in the list of aquatic animal diseases by the OIE. DIV1 is characterized by rapid transmission, a broad host range, and high mortality rate causing crustaceans, but there are no effective drugs for prevention and treatment to diseases causing by DIV1. Therefore, establishing a method with strong specificity, simple operation, rapid results and suitable for large-scale clinical diagnosis is of great significance.

Currently diagnostic methods for DIV1 include clinical diagnosis, histopathological diagnosis, and molecular biological diagnosis. In clinical diagnosis, crustaceans infected with DIV1 exhibit a various of symptoms, which cannot serve as a unified standard for identification. After DIV1 infection, typical disease symptoms are not present, and the symptoms are similar to those caused by pathogens such as WSSV, IHHNV and *Enterocytozoon hepatopenaei* (EHP) (Leu et al., 2009; Yu et al., 2021; Govindasamy et al., 2023), making it impossible to accurately identify DIV1 solely through clinical diagnosis. In histopathological diagnosis, tissue sectioning and electron microscopy techniques have been applied for the preliminary identification of DIV1, but these methods are complex to operate, require high standards for equipment and reagents, and have lower accuracy, making them unsuitable for large-scale clinical diagnosis and only serving as auxiliary examination methods.

Molecular biological diagnostic techniques are the mainstream methods for DIV1 detection. Several methods have established for DIV1 detection including situ hybridization (ISH), polymerase chain reaction (PCR) technology, loop-mediated isothermal amplification (LAMP), and recombinase polymerase amplification (RPA) technology. In 2019, ISH technology established by Qiu et al. (2019) and digoxigenin-labeled *in situ* hybridization (ISDL) established by Chen et al. (2019), which can detected the distribution of DIV1, but cannot quantitied the copies number of DIV1. Chen et al. (2020) and Huang et al. (2022) developed real-time RPA assay for rapid detection of DIV1. The detection limit of the two real-time RPA assay was 2.3 × 10^1^ copies/μL and 11 copies/μL within 20 min at 39°C, respectively. In 2023, Chen et al. (2023) constructed a living magnetotactic microrobot based on bacteria with a surface-displayed CRISPR-Cas12a system for DIV1 detection, and this detection method can detected 8 copies/μL in about 25 min at 37°C. In 2023, Li et al. (2023) established a LAMP method that is sensitive, specific, and rapid, capable of completing detection within 40 min at 40–68°C in the laboratory. The lowest detection limit of the LAMP method is 10^3^ copies/reaction, but it can easily lead to in false positives in DIV1 detection.

The OIE recommends the use of more sensitive and specific Nest-PCR and real-time PCR detection methods for detecting DIV1. Qiu et al. (2017) designed primers based on the ATPase gene sequence of SHIV and established a Nest-PCR method. The lowest detection limit of the Nest-PCR for DIV1 is 36 fg, but it is not conducive to rapid clinical detection and is highly susceptible to aerosol contamination in the laboratory environment leading to false-positive results. To date, two types of real-time PCR methods for detecting DIV1 (Taqman probe method and SYBR Green I method) have been established. Qiu et al. (2018, 2020) designed Taqman probe qPCR detection methods targeting the ATPase gene and the MCP gene of DIV1. The sensitivities of the two methods is 4 copies/reaction and 1.2 copies/reaction, respectively. Gong et al. (2021) also developed a qPCR method based on the ATPase gene, with a sensitivity of 19 copies/reaction. Xu et al. (2023) have reported the development of SYBR Green I-based real-time PCR methods for detection of DIV1. Sensitivity analysis revealed that the real-time PCR could efficiently detect DIV1 DNA as low as 62 copies/μL within 35 cycles. However, the limit of detection of real-time PCR established in the present study is 10 copies/μL within 34 cycles. The real-time PCR method we established has better sensitivity than this method. The Taqman probe qPCR method has high equipment requirements and expensive probe designed, while the SYBR Green I qPCR method is relatively low cost, highly specific, and capable of meeting the requirements for clinical application. Due to the fact that the established methods for detecting DIV1 are main used to different shrimp species, there are certain differences in the sensitivity of these methods.

The MCP protein is the main structural protein of the iridovirus capsid. Biological information analysis by Tidona et al. (1998) to the MCP protein of the members of the family iridoviruses, it is believe that the MCP protein is a suitable target for studying the evolution of iridoviruses, as it contains multiple highly conserved structural domains and its amino acid diversity is sufficient to distinguish between iridovirus species. In addition, the sequence of DIV1 MCP gene has been identified as highly consistent with the sequences of the MCP gene of other iridoviruses in the ninth ICTV report. Therefore, the MCP gene is often used as a target gene for detecting DIV1.

In conclusion, the research results shown that the real-time PCR targeting the MCP gene to DIV1 detection is a strong specificity, high sensitivity, and repeatable and reliable method. It provides a strong foundation for the clinical diagnosis, epidemiology investigation and monitoring of DIV1.

## Data Availability

The original contributions presented in the study are included in the article/supplementary material, further inquiries can be directed to the corresponding authors.

## Footnotes

^1^http://blast.ncbi.nlm.nih.gov/Blast.cgi
